# Comparison of cine and real-time cardiac MRI in rhesus macaques

**DOI:** 10.1038/s41598-021-90106-9

**Published:** 2021-05-21

**Authors:** Amir Moussavi, Sophie Mißbach, Claudia Serrano Ferrel, Hasti Ghasemipour, Kristin Kötz, Charis Drummer, Rüdiger Behr, Wolfram-Hubertus Zimmermann, Susann Boretius

**Affiliations:** 1 Functional Imaging Laboratory, German Primate Center, Leibniz Institute for Primate Research, Göttingen, Germany; 2 DZHK (German Center for Cardiovascular Research), Partner Site, Göttingen, Germany; 3 Platform Degenerative Diseases, German Primate Center, Leibniz Institute for Primate Research, Göttingen, Germany; 4 Institute of Pharmacology and Toxicology, University Medical Center, Göttingen, Germany; 5 Johann-Friedrich-Blumenbach Institute for Zoology and Anthropology, University of Göttingen, Göttingen, Germany

**Keywords:** Translational research, Cardiovascular biology

## Abstract

Cardiac MRI in rhesus macaques, a species of major relevance for preclinical studies on biological therapies, requires artificial ventilation to realize breath holding. To overcome this limitation of standard cine MRI, the feasibility of Real-Time (RT) cardiac MRI has been tested in a cohort of ten adult rhesus macaques using a clinical MR-system. In spite of lower tissue contrast and sharpness of RT-MRI, cardiac functions were similarly well assessed by RT-MRI compared to cine MRI (similar intra-subject repeatability). However, systematic underestimation of the end-diastolic volume (31 ± 9%), end-systolic volume (20 ± 11%), stroke volume (40 ± 12%) and ejection fraction (13 ± 9%) hamper the comparability of RT-MRI results with those of other cardiac MRI methods. Yet, the underestimations were very consistent (< 5% variability) for repetitive measurements, making RT-MRI an appropriate alternative to cine MRI for longitudinal studies. In addition, RT-MRI enabled the analysis of cardio-respiratory coupling. All functional parameters showed lower values during expiration compared to inspiration, most likely due to the pressure-controlled artificial ventilation. In conclusion, despite systematic underestimation of the functional parameters, RT-MRI allowed the assessment of left ventricular function in macaques with significantly less experimental effort, measurement time, risk and burden for the animals compared to cine MRI.

## Introduction

Cardiovascular diseases, in particular coronary artery disease and heart failure, are the leading causes of death in Europe^1^. Translation of promising novel treatment approaches into clinical application often requires animal models very closely resembling humans. In this context, non-human primates (NHPs) have become of great interest for cardiac research^2^. For instance, studies on treatment of post-infarction heart failure by stem cell-based approaches have utilized the close immunological and genetic similarities between humans and NHPs^3–7^.

Monitoring treatment effects and disease progression requires a robust and reliable assessment of left ventricular function. The current gold standard in human cardiac MRI is cine MRI, an ECG-gated gradient echo sequence^8–10^. The required conscious breath-holding during data acquisition, however, can be impossible in many situations. Thus, artificial ventilation with anesthesia has been used for instance for cardiac MRI in neonates, infants and small children^11, 12^.

In contrast to the cine MRI, Real-Time (RT) MRI can be performed without breath holding. By reducing the number of acquired data points, an image can be obtained within about 20 ms^13–16^. The currently proposed methods of RT-MRI differ regarding their steady state of magnetization (spoiled or balanced SSFP), acquisition scheme (Cartesian, radial or spiral), and image reconstruction algorithms. Commonly used are parallel imaging techniques such as GRAPPA^13^, SENSE^14^, and regularized nonlinear inversion (NLINV)^15^.

The feasibility of RT-MRI for cardiac MRI in NHPs is, however, largely unknown. As in infants and young children^11, 12^, the current standard of cardiac MRI in NHPs is to perform “forced” breath holding during artificial ventilation^3, 17–20^. Therefore, RT-MRI may be an alternative and, together with the significant reduction of measurement time, this method may reduce additional risks and burdens for the animal. Rather fast spontaneous breathing and high heart rates (breathing/heart rate: mouse 60/500 bpm^21^, rhesus macaque 37/120 bpm^17^, on average) are, however, particular challenges of RT-MRI in these small animals and the potential bias in quantitative analyses by substantial under sampling needs to be evaluated.

In adult humans, the few studies comparing cine MRI and RT-MRI have mostly reported similar results of left ventricular function obtained with both methods^22–25^. Since cine MRI requires breath holding, comparative studies in children were limited to an age not younger than seven years^26^, but reported likewise similar results.

The aim of this study was to evaluate the capability of RT-MRI for assessing the cardiac function in NHPs and to compare the information obtained by this method with that of cine MRI. This was achieved by measuring each animal twice within few weeks and parameters of left ventricular function were independently extracted for both cine MRI and RT-MRI by two experienced observers. Intra-subject repeatability, inter-observer agreement and systematic differences between the two acquisition methods were evaluated. Moreover, since RT-MRI allowed for the analysis of every single heartbeat, left ventricular function parameters were monitored over at least six heartbeats while the animal was continuously breathing.

## Results

### Left ventricular function measured by cine MRI

Parameters of the left ventricular function and mass obtained by cine MRI (short-axis ECG-gated segmented Cartesian FLASH acquired during expiratory breath holds) at the first (test) and the second study time point (retest) are summarized in Table 1.

**Table 1:** Parameters of the left ventricular function assessed by cine MRI.

Parameter		Observer #1			Observer #2			ICC Inter-observer
		Test	Retest	ICC Intra-subject	Test	Retest	ICC Intra-subject	
LVM (g)	Mean	12.5 ± 2.7	12.6 ± 2.9	0.96	14.2 ± 2.7	14.2 ± 2.6	0.98	0.98
	Mean Difference	− 0.2 ± 1.0			0 ± 0.8			
LVWM (g)	Mean	11.8 ± 2.5	12.0 ± 2.8	0.96	13.4 ± 2.6	13.3 ± 2.5	0.97	0.97
	Mean difference	− 0.1 ± 1.0			0 ± 0.8			
PM (g)	Mean	0.6 ± 0.2	0.7 ± 0.2	0.92	0.9 ± 0.2	0.9 ± 0.2	0.94	0.68
	Mean difference	0 ± 0.1			0 ± 0.1			
EDV (ml)	Mean	14.8 ± 2.7	13.5 ± 2.4	0.93	15.3 ± 2.8	13.8 ± 2.3	0.95	0.99
	Mean difference	1.3 ± 1.4			1.4 ± 1.1			
ESV (ml)	Mean	6.3 ± 1.5	5.9 ± 1.6	0.82	6.9 ± 1.6	6.3 ± 1.5	0.91	0.98
	Mean difference	0.3 ± 1.2			0.6 ± 0.9			
SV (ml)	Mean	8.6 ± 1.5	7.5 ± 1.2	0.84	8.3 ± 1.4	7.5 ± 1.0	0.90	0.97
	Mean difference	1.0 ± 1.0			0.8 ± 0.8			
EF (%)	Mean	58 ± 5	57 ± 6	0.58	55 ± 4	55 ± 5	0.64	0.94
	Mean difference	2 ± 6			0 ± 5			
CO (l/min)	Mean	0.9 ± 0.2	0.8 ± 0.1	0.74	0.8 ± 0.2	0.8 ± 0.1	0.43	0.94
	Mean difference	0.1 ± 0.1			0.1 ± 0.1			

All data given as arithmetic mean and standard deviation (n = 10). Mean difference was defined as the individual differences between test and retest experiment. ICC: Intraclass Correlation Coefficient; LVM: Left Ventricular Mass; LVWM: Left Ventricular Wall Mass; PM: Papillary Muscle Mass; EDV: End-Diastolic Volume; ESV: End-Systolic Volume; SV: Stroke Volume; EF: Ejection Fraction; CO; Cardiac Output.

Left ventricular wall mass (LVWM; median: 12.3, range: 8.0–18.1 g), end-diastolic volume (EDV; median: 13.9, range: 10.3–19.2 mL), end-systolic volume (ESV; median: 6.5, range: 3.7–8.8 mL) and stroke volume (SV; median: 8.0, range: 5.6–11.6 mL) varied widely between the animals, but showed an excellent intra-subject repeatability (Table 1 and Supplementary Fig. 1). In contrast, the ejection fraction (EF) appeared less variable between the animals (median: 56.3, range 45.8–66.5%) but showed only a moderate intra-subject repeatability. On average, LVWM and EF exhibited no systematic differences between repetitive experiments, whereas slightly lower volumes (EDV, ESV and SV) were observed in retest experiments as supported by the Bland–Altman plots (Supplementary Fig. 1). All evaluated parameters exhibited an excellent inter-observer repeatability (range: 0.82–0.98). Only the papillary muscle mass (PM) showed a lower inter-observer agreement.

Including or excluding the papillary muscles from the myocardium did not significantly change the absolute values of cardiac function. The PM was on average 0.8 g (± 0.2) which corresponded to about 5.5% of the total LVM (Range: 2.1–8.1%). Including the papillary muscle from the myocardium, led, as expected, to a decrease in the calculated EDV, ESV and SV of about 6% (± 1.4), 10% (± 2.9) and 3% (± 2.3), respectively. In contrast, the EF increased by about 3% (± 1.9).

### Comparison of cine and real-time MRI

#### Image quality of cine and RT-MRI

**Figure 1:**
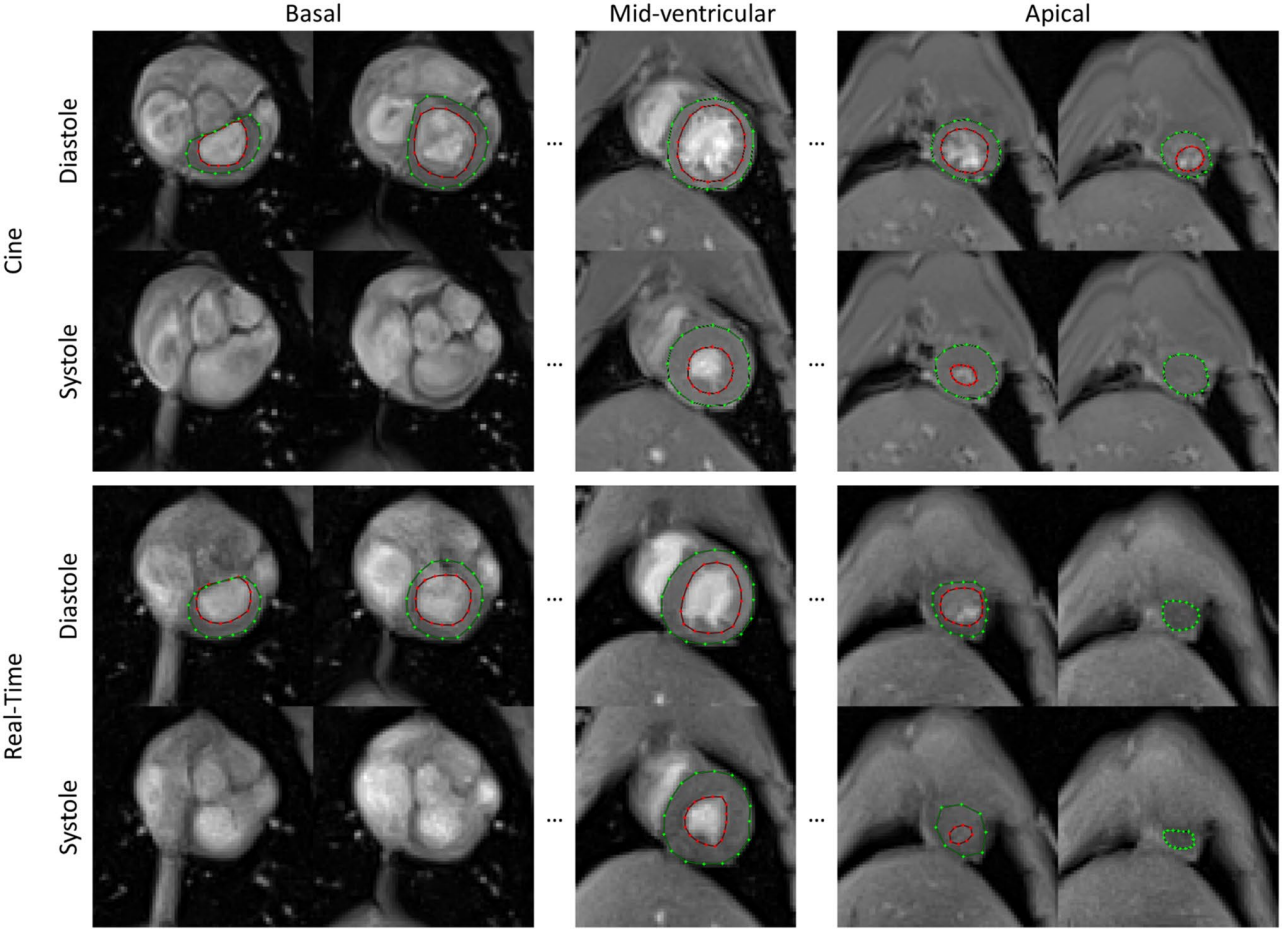
Qualitative comparison of cine MRI and RT-MRI. Compared to cine MRI (top), RT-MRI (bottom) exhibited a reduced image sharpness making the demarcation of the papillary muscles and the trabeculae challenging. Epicardial and endocardial delineations are marked in green and red, respectively. All images were acquired during expiration.

RT-MRI allowed for mostly artifact-free data acquisition while the animals were continuously breathing. Figure 1 illustrates the image quality achieved by cine MRI and RT-MRI of selected slices of the left ventricle in both diastolic and systolic phase. Compared to cine MRI, RT-MRI revealed a slightly lower tissue contrast and a reduced image sharpness. For instance, the ratio of the signal intensities of blood and myocardium was about 10% lower in RT-MRT compared to cine MRI (t-test, *p* < 0.01). The lower tissue contrast hampered in particular the definition of the most basal and the most apical slice of the heart. Moreover, the lower image sharpness led to a more challenging segmentation of the papillary muscles and the trabeculae (arrows in Supplementary Fig. 2). As shown exemplarily for a mid-ventricular slice (Supplementary Fig. 2), the signal profile of RT-MRI appeared smoother and Gaussian shaped compared to the signal profile of cine MRI.

The time needed for the myocardial segmentation of RT-MRI data sets was on average five times longer compared to cine MRI, mainly due to the larger number of separately segmented heart cycles. In addition, the lower tissue contrast and image sharpness as well as the lack of appropriate segmentation software prolonged the segmentation time.

#### Quantitative comparison of the heart function parameters

Table 2 summarizes the volumetric and functional parameters of the left ventricle for the respective respiratory periods (inspiration and expiration) obtained by RT-MRI. Similar to cine MRI, significant inter-subject variability and excellent to good intra-subject repeatability were observed for all volumetric parameters of the left ventricle. The EF showed only a good to moderate intra-subject repeatability. The corresponding correlation and Bland–Altman plots are shown in Supplementary Fig. 3. In addition, and in line with the results of cine MRI, an excellent inter-observer agreement (range: 0.87–0.99) was achieved for all assessed volumetric and functional parameters obtained by RT-MRI.

**Table 2:** Parameters of the left ventricular function assessed by Real-Time MRI.

Parameter			Observer #1			Observer #2			ICC Inter-observer
			Test	Retest	ICC Intra-subject	Test	Retest	ICC Intra-subject	
LVWM (g)	Expiration	Mean	14 ± 3	15 ± 3	0.96	14 ± 3	14 ± 3	0.90	0.97
		Mean difference	0 ± 1			0 ± 2			
	Inspiration	Mean	16 ± 4	16 ± 3	0.97	15 ± 4	14 ± 3	0.89	0.96
		Mean difference	0 ± 1			1 ± 2			
EDV (ml)	Expiration	Mean	11 ± 3	9 ± 3	0.96	11 ± 2	9 ± 2	0.94	0.96
		Mean difference	1 ± 1			1 ± 1			
	Inspiration	Mean	12 ± 3	11 ± 3	0.92	13 ± 3	12 ± 3	0.94	0.99
		Mean difference	1 ± 2			1 ± 1			
ESV (ml)	Expiration	Mean	5 ± 1	4 ± 1	0.88	6 ± 1	5 ± 1	0.89	0.97
		Mean difference	1 ± 1			1 ± 1			
	Inspiration	Mean	6 ± 1	5 ± 1	0.71	6 ± 1	6 ± 1	0.88	0.93
		Mean difference	0 ± 1			1 ± 1			
SV (ml)	Expiration	Mean	6 ± 2	5 ± 2	0.92	5 ± 1	4 ± 1	0.80	0.93
		Mean difference	1 ± 1			0 ± 1			
	Inspiration	Mean	7 ± 2	6 ± 2	0.91	7 ± 2	6 ± 2	0.92	0.96
		Mean difference	1 ± 1			1 ± 1			
EF (%)	Expiration	Mean	52 ± 8	52 ± 7	0.75	45 ± 6	46 ± 5	0.46	0.90
		Mean difference	1 ± 6			− 1 ± 7			
	Inspiration	Mean	55 ± 7	55 ± 8	0.80	52 ± 6	52 ± 6	0.84	0.87
		Mean difference	0 ± 7			− 1 ± 4			

All data given as arithmetic mean and standard deviation (n = 10). Mean difference was defined as the individual differences between test and retest experiment. ICC: Intraclass Correlation Coefficient; LVWM: Left Ventricular Wall Mass; EDV: End-Diastolic Volume; ESV: End-Systolic Volume; SV: Stroke Volume; EF: Ejection Fraction.

**Figure 2:**
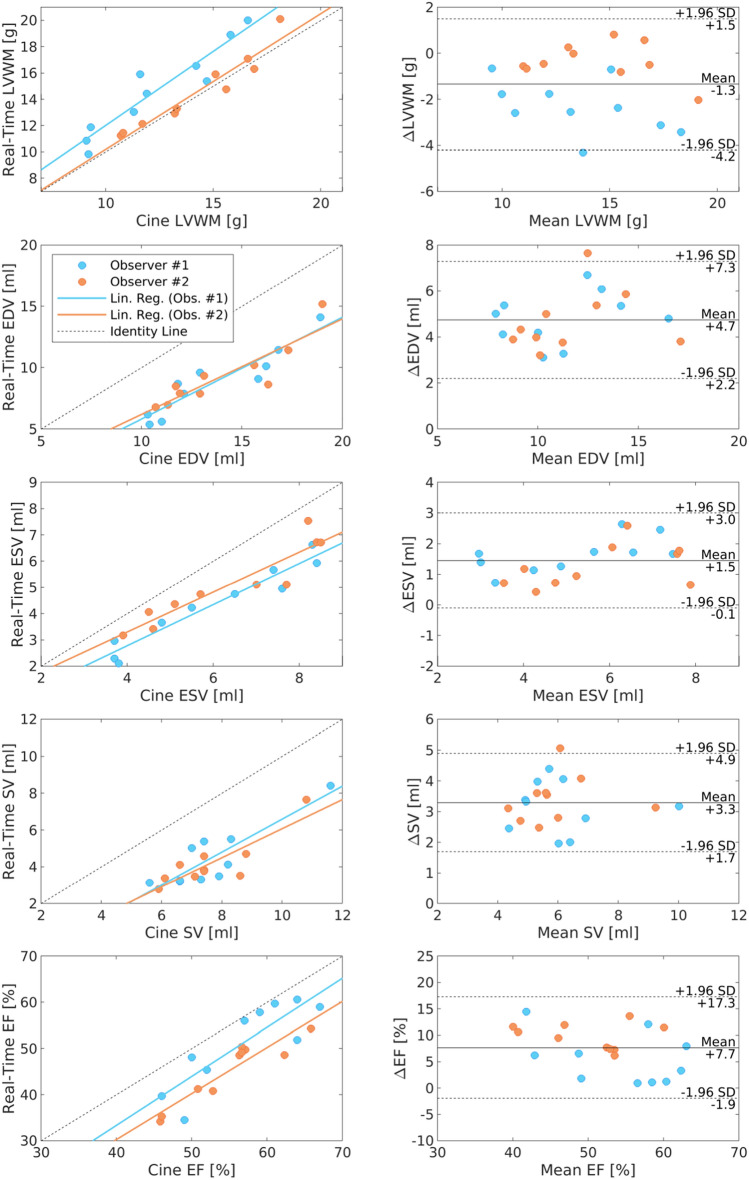
Comparison of cine MRI and RT-MRI. Correlation and Bland–Altman plots of LVWM, EDV, ESV, SV and EF comparing cine MRI and RT-MRI of the first study time point (test). RT-MRI significantly underestimates volumetric parameter by 20% to 40% when taking cine MRI as gold standard. However, regression analysis comparing estimated parameter by RT-MRI and cine MRI revealed a strong correlation. LVWM: Left Ventricular Wall Mass; EDV: End-Diastolic Volume; ESV: End-Systolic Volume; SV: Stroke Volume; EF: Ejection Fraction.

To compare cine MRI and RT-MRI, only real-time data acquired during expiration was chosen and papillary muscles were excluded from the myocardium. Absolute and percentage differences between parameters acquired by cine MRI and by RT-MRI are summarized in Table 3. In general, RT-MRI significantly underestimates all volumetric parameters (EDV by 31 ± 9%, ESV by 20 ± 11% and SV by 40 ± 12%). The resulting EF was, however, only slightly lower (13 ± 9%) compared to the EF estimated by cine MRI. In spite of the volumetric underestimation, RT-MRI slightly overestimated the LVWM by about 13%. Figure 2 and Supplementary Fig. 4 illustrate the systematic differences between cine MRI and RT-MRI for test and retest experiments, respectively. Test–retest repeatability yields a high degree of consistency for the systematic volumetric underestimations of RT-MRI.

**Table 3:** Difference between cine MRI and Real-Time MRI.

Parameter		Observer #1				Observer #2			
		Absolute		Percentage		Absolute		Percentage	
		Test	Retest	Test	Retest	Test	Retest	Test	Retest
LVWM (g)	Mean Difference	− 2 ± 1	− 3 ± 1	− 20 ± 7	− 23 ± 10	− 1 ± 1	− 1 ± 1	− 6 ± 8	− 4 ± 9
	Test–Retest Diff	0 ± 1		3 ± 11		0 ± 1		− 2 ± 10	
EDV (mL)	Mean	4 ± 2	4 ± 1	29 ± 11	32 ± 10	5 ± 1	4 ± 1	31 ± 7	33 ± 7
	Mean difference	0 ± 1		− 3 ± 9		0 ± 1		− 2 ± 8	
ESV (mL)	Mean	1 ± 1	1 ± 1	19 ± 13	25 ± 9	1 ± 1	1 ± 1	15 ± 10	19 ± 8
	Mean difference	0 ± 1		− 5 ± 13		0 ± 1		− 4 ± 9	
SV (mL)	Mean	3 ± 1	3 ± 1	36 ± 13	37 ± 15	4 ± 1	3 ± 1	43 ± 8	43 ± 10
	Mean difference	0 ± 1		− 1 ± 11		0 ± 1		0 ± 11	
EF (%)	Mean	6 ± 4	5 ± 6	10 ± 9	8 ± 10	10 ± 3	9 ± 5	18 ± 5	16 ± 9
	Mean difference	1 ± 6		2 ± 9		1 ± 5		2 ± 9	

All data given as arithmetic mean and standard deviation (n = 10). Mean difference and test–retest difference were defined as the individual differences between cine MRI and Real-Time MRI, and between test and retest experiment, respectively. LVWM: Left Ventricular Wall Mass; EDV: End-Diastolic Volume; ESV: End-Systolic Volume; SV: Stroke Volume; EF: Ejection Fraction.

### Effect of the respiratory phase on left ventricular function

**Figure 3:**
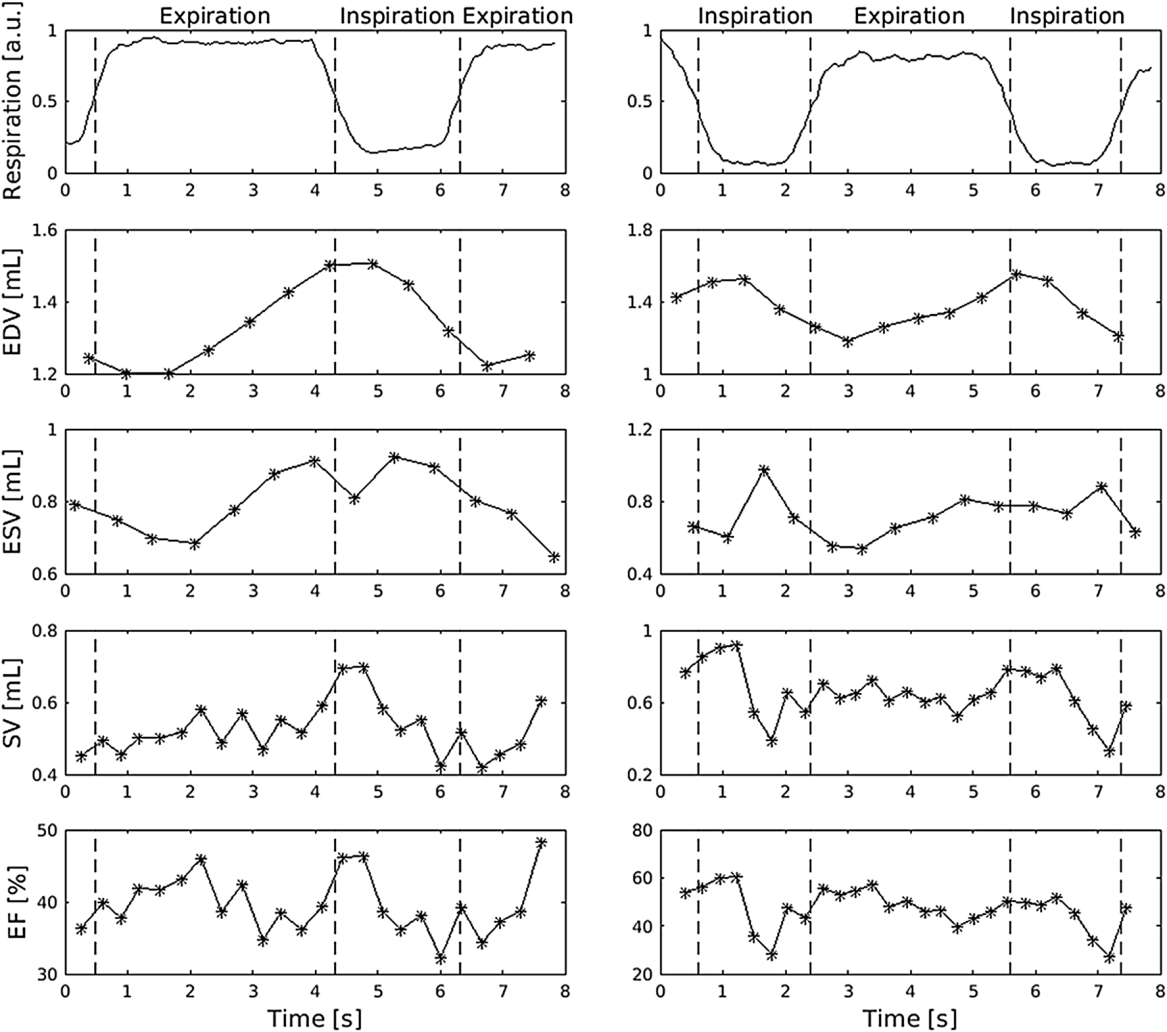
Function-time curve over consecutive heartbeats. The signal-time curve of the respiratory signal (normalized by mean of maximum for expiration and minimum for inspiration) and the extracted functional parameter (EDV, ESV, SV and EF) of a Real-Time experiment over consecutive cardiac cycles were exemplarily plotted for a mid-ventricular slice of two different animals. Both EDV and ESV systematically show a periodical behavior leading to highest volumes in inspiration and lowest at expiration. Although EDV and ESV show fluctuations up to 30% in each cardiac cycle the estimated EF has only a minor fluctuation up to 10%. EDV: End-Diastolic Volume; ESV: End-Systolic Volume; SV: Stroke Volume; EF: Ejection Fraction.

Using RT-MRI we were able to analyze the cardiac function for every heartbeat and respiratory phase separately. Figure 3 shows exemplarily the EDV, ESV, SV, and EF for consecutive cardiac cycles obtained from a mid-ventricular slice of two different animals. The first graph indicates the respective respiration phases (normalized signal of the lung-liver interface). Both, EDV and ESV, revealed a consecutive increase during inspiration and a decrease during expiration, still continuing when the expiration already started. Whereas the time curve of EDV appeared rather smooth, the ESV revealed, albeit only very few, jumps during inspiration changing its value from one heartbeat to the other by sometimes more than 50%.

**Figure 4:**
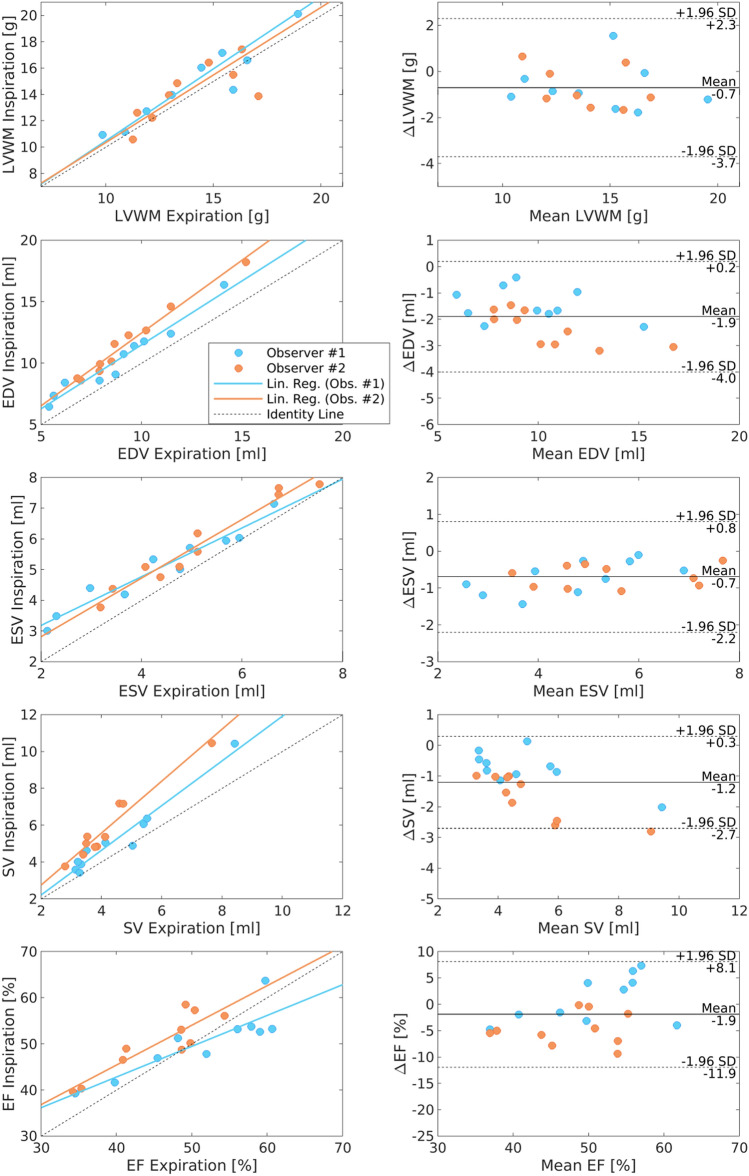
Comparison of inspiratory and expiratory ventricular function. Correlation and Bland–Altman plots of LVWM, EDV, ESV and EF for inspiration and expiration obtained by RT-MRI of the first study time point (test). Volumetric parameters (EDV, ESV and SV) were increased by at least 20% during inspiration compared to expiration. Additionally, a slight increase in EF was observed during inspiration. Nevertheless, a strong correlation between the two respiration phases exists. LVWM: Left Ventricular Wall Mass; EDV: End-Diastolic Volume; ESV: End-Systolic Volume; SV: Stroke Volume; EF: Ejection Fraction.

By analyzing the entire left ventricle, EDV and ESV exhibited about 10–20% higher values in inspiration compared to expiration while the SV increased by about 40%. In contrast, EF showed only a small increase of less than 10% while LVWM remained almost constant. Beside the difference in absolute values, EDV, ESV, SV, and EF showed a strong relationship between the two respiration phases as illustrated in Fig. 4 (test) and Supplementary Fig. 5 (retest).

## Discussion

This study compares cine MRI and RT-MRI for assessing left cardiac ventricular function in healthy rhesus macaques, a non-human primate species of significant importance in preclinical research on biological therapies. In addition to demonstrating the feasibility of RT-MRI in animals with higher heart rate (~ 105 bpm) and much smaller heart size (with a total heart size of ~ 40 g roughly 10% of the human heart), we showed a similar data quality of RT-MRI and cine MRI. Particular advantages of RT-MRI were the possibility of beat-by-beat image analysis and markedly shorter acquisition time. Another clear advantage of RT-MRI is that it does not require breath holding alleviating the need for artificial ventilation. This significantly reduces the preparation and measurement time, the duration of anesthesia and therefore the potential risk and burden of the animal. With the chosen protocols, we could reduce the acquisition time, in comparison to cine MRI, by a factor of more than two. Taking the recovery time between two breath holds into account, which may be particularly relevant in cardiac disease models, the acceleration factor increases even more. In fact, it was possible to limit the total scan time including localizer and functional data set to about five minutes per animal. However, these benefits were achieved at the expense of a slightly reduced sharpness of the image and a lower tissue contrast. This fact made it more challenging to identify the most basal and the most apical slice of the heart. Moreover, the achieved image quality hindered an automatic segmentation of myocardial contours with the current available software tools. The lower image quality of RT-MRI, however, did not lead to a reduced repeatability of the method. In fact, cine MRI and RT-MRI revealed very similar intra-subject repeatability and an excellent inter-observer agreement. In particular, EDV was best repeatable, but even the lower repeatability of ESV, SV and EF was very much in line with those reported in human studies^23, 27, 28^. The almost similar LVWM values suggest that physiological differences may dominate the difference observed between the two study time points. Moreover, the only two non-human primate studies having so far addressed the repeatability of cardiac function measured by cine MRI presented very similar results^3, 17^.

The direct comparison of cine MRI and RT-MRI revealed a strong linear relationship for the calculated parameters of cardiac function, similar to what has been reported in humans^22–24^. Although exhibiting the strongest correlation for the two methods, volumetric parameters (EDV, ESV and SV) were systematically underestimated by approximately 20 to 40% when using RT-MRI. This is in contrast to previous human studies^22, 23, 29^ and most likely caused by the required regularization during image reconstruction, the intrinsic characteristics of the radial point-spread-function which may be more prominent in the smaller and faster beating heart of a rhesus macaque and inaccuracies in the delineation of endocardial contours of the myocardium. This underestimation was, however, rather consistent. The two methods revealed almost identical differences between the two study time points (Table 3) justifying group comparison and follow up measurements, when identical MR and reconstruction parameters are used.

Therewith, RT-MRI can be a valuable alternative in cases where the use of other real time cardiac imaging methods such as ultrasound may be hampered by model related particularities such as surgical interventions and a study design requiring cardiac MRI for transmissibility to humans. Moreover, the extension of the proposed method to parametric imaging such as T1 mapping and contrast-agent based perfusion imaging will significantly widen its scope of application.

A limitation of this study is the exclusive use of spoiled segmented FLASH instead of balanced steady-state free precession (bSSFP), a MR sequence commonly used in cardiac MRI of adult humans. However, bSSFP sequences are, especially at higher magnetic fields and spatial resolution used here, particularly sensitive to differences in magnetic susceptibility and to magnetic field inhomogeneities which often lead to disturbing banding artifacts^30^. In addition, in rhesus macaques (and infants) the required high spatiotemporal resolution of RT-MRI restricted the use of bSSFP due to the higher specific absorption rates (SAR) of the sequence.

One of the advantages of RT-MRI is the possibility to analyze individual heartbeats and therefore to study the interaction between respiration and cardiac function. It has been shown that normal breathing^31–36^ and respiratory maneuvers^33–35^ can significantly influence the cardiac flow, volumes and function. However, the exact underlying mechanism is not clear yet. RT-MRI offers the unique opportunity to investigate these effects during free and artificial breathing. In our experiments (constant-pressure artificial ventilation) higher volumetric values were observed during inspiration, while in case of free breathing EDV, ESV and SV revealed the highest values during expiration (^36, 37^ and our own experiments, data not shown). This is most likely due to the fact that in normal breathing the inspiration goes along with a reduction of chest pressure, whereas in case of artificial ventilation the breathing air is actively pressed into the airways leading to a pressure increase on the vascular system.

Due to the continuously changing of EDV and ESV during breathing, we strongly recommend the inclusion of more than one heart cycle in the quantitative analysis of RT-MRI. Based on this study we suggest acquiring cardiac Real-Time data continuously over at least two breathing periods. The respective heart cycles can then be selected based on their position in the breathing cycle (determined from the signal-time curve of the lung-liver interface) which will allow a more robust comparison between different animals.

Another, and for quantitative group analyses probably even more suitable approach, could be the use of self-gating algorithms utilizing, for instance, signal and phase information of the central data point of each k-space line. Unfortunately, the best sampling schema for RT-MRI is not also the best for self-gating. Thus, this would exceed the scope of the manuscript and will be the subject of another study.

In conclusion, Real-Time cardiac MRI allows for the assessment of left ventricular cardiac function of NHPs. Compared with cine MRI, it significantly reduces the potential burden of the animal. The absolute values of cardiac parameters differed between cine MRI and RT-MRI, which hampers a direct comparison across sites and studies. However, comparisons within each of the two methods will most likely reveal very similar results.

In addition, further optimization of tissue contrast and sharpness, as well as development of automatic segmentation tools are required to speed up the currently rather time-consuming data analysis. The lack of appropriate software to automatically or semi-automatically quantify the cardiac function using RT-MRI is currently probably the biggest barrier in bringing it to a broader application.

## Methods

### Animals

A cohort of 10 adult, healthy rhesus macaques (*macaca mulatta*, mean age: 7.5 ± 2.2 year, including 5 males) was included in this study. All monkeys were purpose-bred, raised and housed according to the standards for macaques of the German Primate Center (Göttingen, Germany). All animal experiments were in full accordance with the ARRIVE guidelines, the German animal welfare law (TierSchG), the regulations for research animals (TierSchVerV) and approved by local authorities (Animal Welfare Service, Lower Saxony State Office for Consumer Protection and Food Safety, license-number 33.9-42502-04-16/2370). Initially, animals were sedated with an intramuscular injection of 7 mg/kg ketamine (100 mg/ml, WDT, Germany) and 0.04 mg/kg medetomidine (Domitor 1 mg/ml, Vetoquinol GmbH, Germany). Anesthesia was maintained via continuous infusion of 10–40 mg/kg/h Propofol-Lipuro 2% (B. Braun Melsungen AG, Germany). The intubated monkeys were pressure-controlled ventilated (Servo Ventilator 900C, Siemens-Elema AB, Sweden) with at least 60% oxygen in the exhalation air in order to keep the O_2_ saturation between 98–100% (measured by pulse oximetry, Nonin 7500FO, Nonin Medical Inc., USA). The CO_2_ concentration in the exhalation air (IntelliVue, Philips, Germany) was maintained below 30% to avoid spontaneous breathing.

All animals were measured twice within an interval of 2 to 15 weeks (mean 7 weeks). While the heart rate under anesthesia (HR, median: 105, range: 79–134 bpm) varied widely between the two study time points (mean difference of 3 ± 11 bpm), the body weights (BW, median: 7.6, range: 5.9–10.2 kg) remained stable (mean difference of 0 ± 0.2 kg).

### MRI

Measurements were performed on a 3T MRI-system (Magnetom Prisma, Siemens Healthineers, Erlangen, Germany) using a 16-channel multipurpose coil (Variety, Noras MRI products, Hoechberg, Germany) for signal detection. The animals were positioned in supine position. Physiological parameters such as heart rate, breathing rate and end-tidal CO_2_ were continuously monitored.

*Cine MRI:* For slice positioning, low-resolution (1.55 × 1.55 × 8 mm^3^) ECG-gated T1-weighted images were conducted during free breathing in 2-chamber, 4-chamber, and short-axis orientation. Subsequently, short-axis cine multi-phase segmented FLASH data sets were acquired using the following parameters: repetition time (TR) = 6.48 ms, echo time (TE) = 2.84 ms, flip angle = 12°, bandwidth = 215 Hz/Px, field of view (FOV) = 132 × 149 mm^2^, acquisition matrix = 128 × 176 (reconstructed to 156 × 176), 5 segments, readout oversampling = 2, spatial resolution = 0.85 × 0.85 mm^2^, slice thickness = 3 mm, slice gap = 0.3—0.6 mm, and 12–14 slices covering the heart from the apex to the base. No parallel imaging was used. Data was continuously recorded over 26 heartbeats and then retrospectively reconstructed to 25–40 phases per cardiac cycle. The number of phases per cardiac cycle ($p$) was set individually for each experiment based on the respective heart rate (HR):

1 $$p= \frac{60}{HR\times TR}\times 2.$$

MR data was obtained during breath holds (15–20 s at average) in the expiratory phase. Including resting time of at least two minutes between two breath-holds the total measurement time of a complete cine dataset was around 30 min. Noteworthy to mention, the pure time of data acquisition for a single slice was on average 15 s.

*RT-MRI:* Similar to the localizing measurements for cine MRI, data sets were acquired in 2-chamber, 4-chamber, and short-axis orientation. This time, however, a low-resolution version of the final RT-MRI protocol was used (spatial resolution = 1.28 × 1.28 × 4 mm^3^, 3 slices, slice gap = 0.8 mm, temporal resolution = 90 ms per slice, 15 repetitions per slice) without any cardiac triggering and breath holding. Real-Time data sets for quantification of left ventricular function were acquired using spoiled radial FLASH with an interleaved ordering scheme: TR = 2.89 ms, TE = 1.92 ms, flip angle = 8°, bandwidth = 1305 Hz/Px, FOV = 128 × 128 mm^2^, base resolution = 128, 13 spokes per frame, nyquist undersampling factor = 15.5, spatial resolution = 0.9 × 0.9 mm^2^, slice thickness 3 mm, slice gap = 0.3–0.6 mm, acquisition time per frame = 37 ms, 210 frames per slice and 12–14 consecutively acquired short-axis slices covering the heart from the apex to the base. The scan time for a single slice was 8.2 s. The number of heart cycles covered by the 210 frames was depended on the individual heart rate and were in the range of about 10 to 15 consecutive cycles per slice. Online reconstruction of Real-Time data sets was carried out using the iterative non-linear approach described by Uecker et al.^38^ and was performed in real time on a graphical processing unit by bypassing the MRI host.

### Assessment of left ventricular function

The left ventricle function of short-axis images was independently evaluated in a blind fashion by two observes with at least two years of experiences in cardiovascular MRI of primates using the freely available software package Segment (Version 2.0 R6435, Medviso, Lund, Sweden)^39^.

For cine MRI end-diastolic and the end-systolic phase were selected as the phases with the highest and lowest blood volume of a mid-ventricular slice, respectively. Endocardial and epicardial contours were manually drawn from base to apex of the heart in both cardiac phases (Fig. 1). According to the guidelines of standardized image interpretation the most basal slice was defined as the slice in which at least 50% of the blood volume was surrounded by myocardium^40^. The most apical slice was defined as the last slice, which includes blood volume. The resulting number of analyzed slices per animals was 10–12.

**Figure 5:**
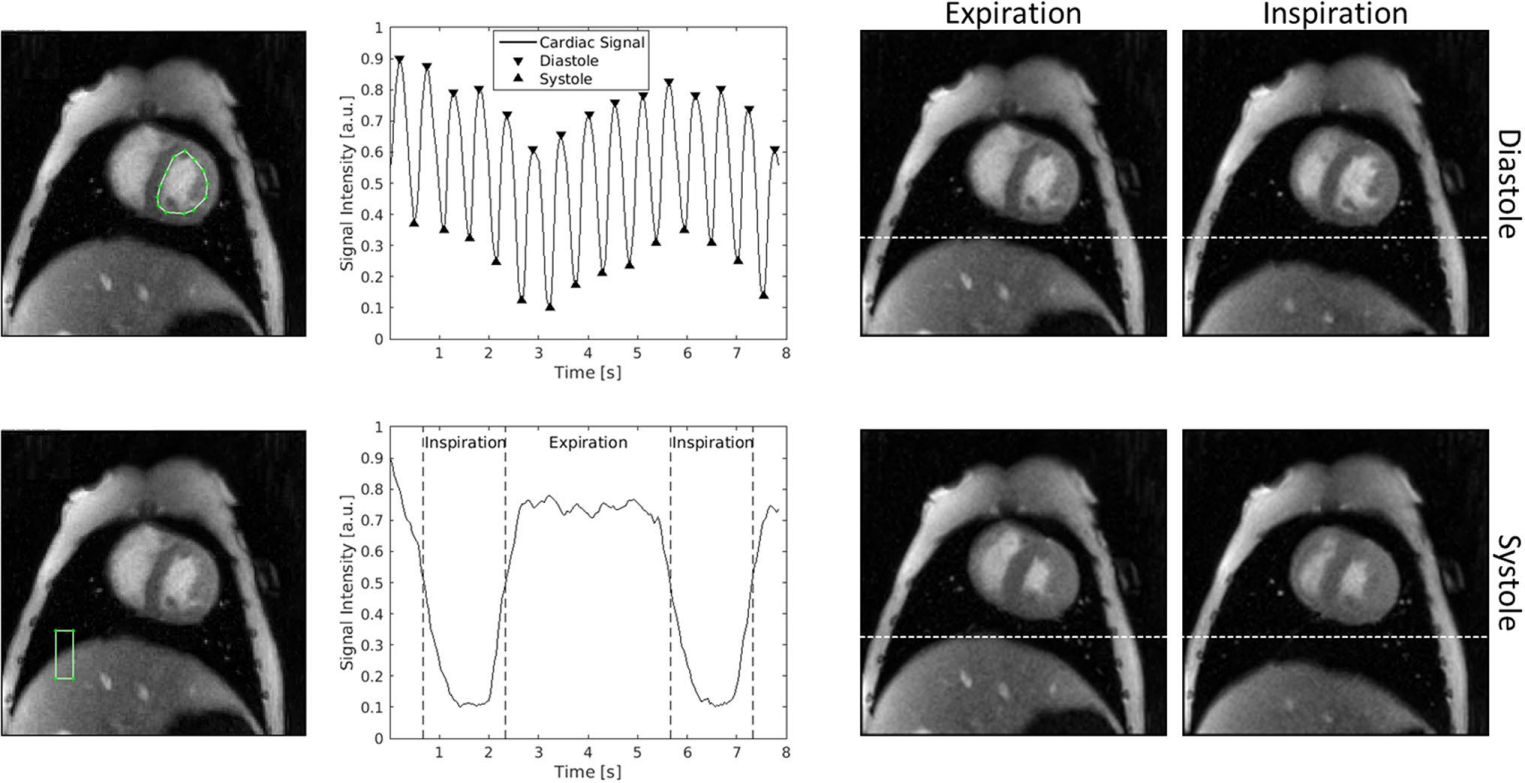
Semi-automatic extraction of cardiorespiratory phases. The cardiorespiratory phase was extracted from signal-time curves of region of interests manually drawn in the left ventricle (top left) and at the lung-liver interface (bottom left). The diastolic and systolic phases (black arrows) were extracted from the signal-time curve of the left ventricle using a peak detection algorithm. The inspiration and expiration are detected based on thresholds defined by the respiratory signal (separated by dashed lines). Representative end-diastolic and end-systolic images obtained during inspiration and expiration are shown on the right. The movement of the liver due to respiration is clearly visible (dashed lines).

In case of RT-MRI data sets were firstly sorted regarding their cardiorespiratory phase using an in-house Matlab script (R2015a, MathWorks, Natick, USA). Therefore, the signal-time curve of manually drawn regions of interest in the left ventricle and at the lung-liver interface were extracted for each slice. The diastolic and systolic phases were selected automatically using a peak detection algorithm. Inspiration and expiration were separated by signal thresholding. Heart cycles in the transition phase of inspiration and expiration were neglected. This procedure is exemplary shown for a mid-ventricular slice in Fig. 5. It should be noted that in RT-MRI slices were acquired sequentially and hence belong to different heart cycles. Therefore, parameters such as the end-diastolic volume (EDV) and the end-systolic volume (ESV) were calculated slice-wise and averaged over all heart cycles, separately for inspiration and expiration phase.

Based on the segmentation of endocardial and epicardial contours on the cine and Real-Time data sets the end-diastolic volume (EDV) and the end-systolic volume (ESV) were calculated using Simpson’s method of disks^41^. The stroke volume (SV), the ejection fraction (EF) and the cardiac output (CO) were defined as the difference between EDV and ESV, the relation of SV and EDV and the product of SV and heart rate (HR), respectively. Additionally, for cine data sets left ventricular wall mass (LVWM) and the papillary mass (PM) were calculated as the average of the corresponding mass in the diastolic and systolic phase. The left ventricular mass (LVM) was determined as the sum of LVWM and PM.

### Statistical analysis

Intra-subject repeatability and inter-observer agreement were calculated using the intraclass correlation coefficient (ICC). Additionally, regression analysis and Bland–Altman plots were used to visualize possible systematic trends. Limits of agreement for repetitive experiments and multi observer were calculated as described by Bland and Altman^42^. All statistics were carried out in Matlab (R2015a, MathWorks, Natick, USA).

## Supplementary Information

Supplementary Information.

## Data Availability

The datasets used and/or analyzed during the current study are available from the corresponding author on reasonable request.
