# Supplementary material for: Comparison of cine and real-time cardiac MRI in rhesus macaques
Source: Sci Rep. 2021 May 21;11:10713. doi: 10.1038/s41598-021-90106-9 (PMC8140156; doi:10.1038/s41598-021-90106-9)

Comparison of Cine and Real-Time Cardiac MRI in Rhesus Macaques

Amir Moussavi[1,2*], Sophie Mißbach[2,3], Claudia Serrano Ferrel[1,4], Hasti Ghasemipour[1], Kristin Kötz[1], Charis Drummer[2,3], Rüdiger Behr[2,3], Wolfram-Hubertus Zimmermann[2,4], Susann Boretius[1,2,5]

[1]Functional Imaging Laboratory, German Primate Center, Leibniz Institute for Primate Research, Göttingen, Germany

[2]DZHK (German Center for Cardiovascular Research), partner site Göttingen, Germany

[3]Platform Degenerative Diseases, German Primate Center, Leibniz Institute for Primate Research, Göttingen, Germany

[4]Institute of Pharmacology and Toxicology, University Medical Center, Göttingen, Germany

[5]Johann-Friedrich-Blumenbach Institute for Zoology and Anthropology, University of Göttingen, Germany

*Corresponding author: Amir Moussavi, German Primate Center, Göttingen, Germany. Tel: +49-551-3851-392. E-Mail: amoussavi@dpz.eu.

Supplementary Figure 1: Intra-subject repeatability of cine MRI. Correlation and Bland-Altman plots of LVWM, EDV, ESV, SV and EF for test and retest measurements revealed a good repeatability of cine MRI. The Bland-Altman plots uncovered no systematic difference between test and retest. LVWM: Left Ventricular Wall Mass; EDV: End-Diastolic Volume; ESV: End-Systolic Volume; SV: Stroke Volume; EF: Ejection Fraction.

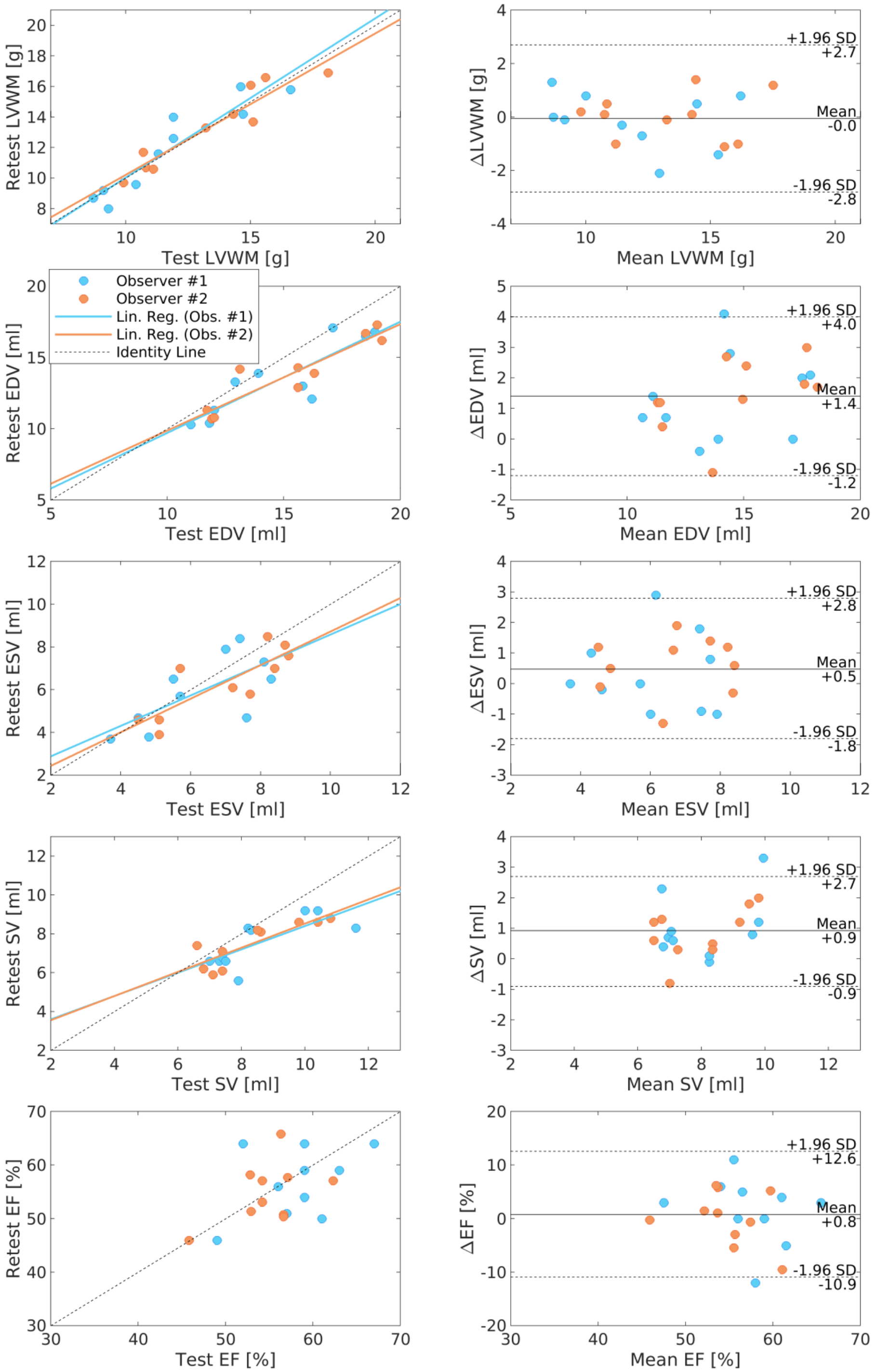

Supplementary Figure 2: Tissue contrast and image sharpness. RT-MRI showed a lower tissue contrast, particular prominent when comparing blood (A, C) and myocardium (B) and a lower image sharpness leading to a more challenging segmentation of the papillary muscles and the trabeculae (arrows). The signal profile (bottom) through both ventricles of a mid-ventricular slice obtained by RT-MRI appeared smoother and Gaussian shaped compared to the signal profile of cine MRI.

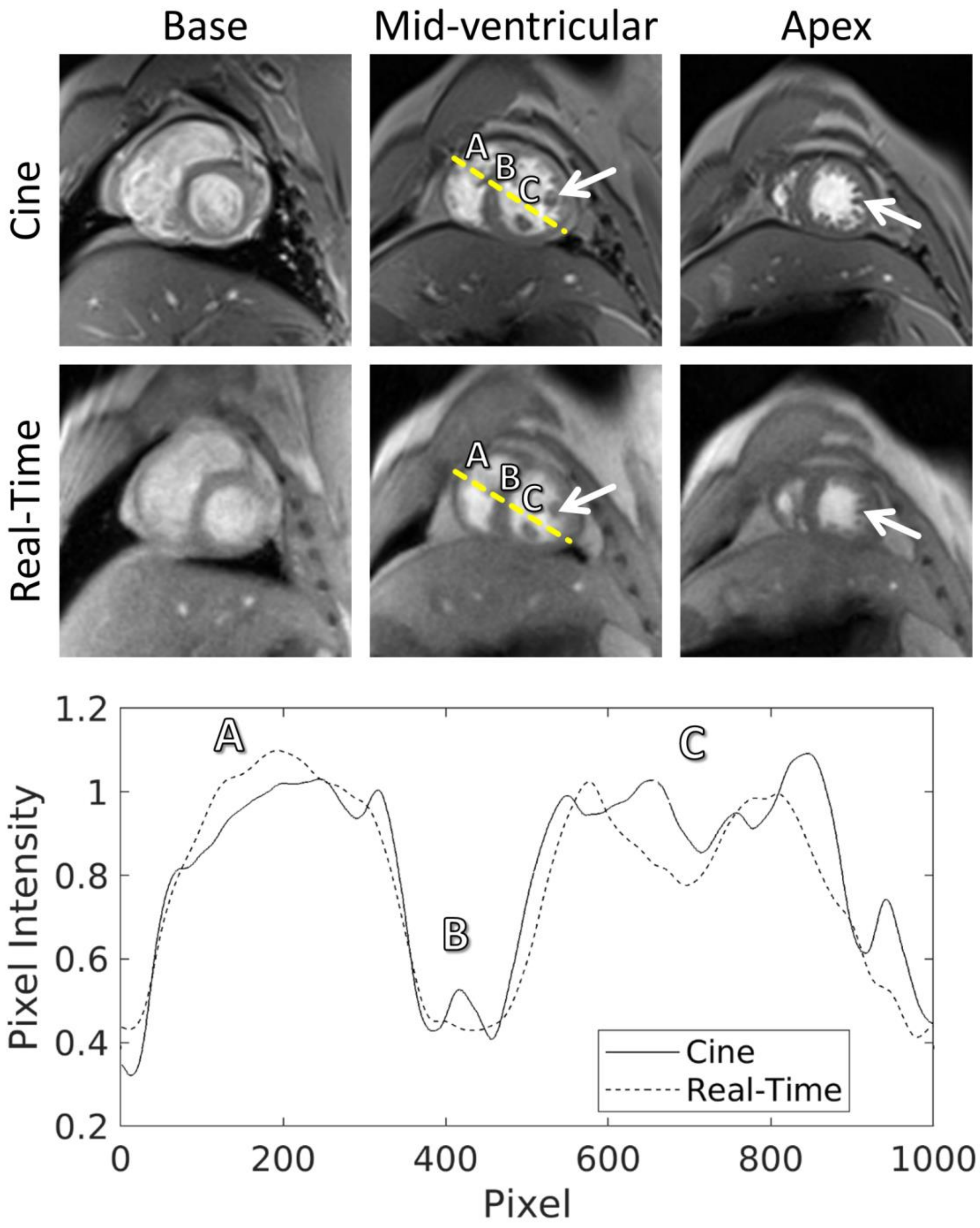

Supplementary Figure 3: Intra-subject repeatability of RT-MRI. Correlation and Bland-Altman plots of LVWM, EDV, ESV, SV and EF for test and retest measurements revealed a good repeatability of RT-MRI. The Bland-Altman plots uncovered no systematic difference between test and retest. LVWM: Left Ventricular Wall Mass; EDV: End-Diastolic Volume; ESV: End-Systolic Volume; SV: Stroke Volume; EF: Ejection Fraction.

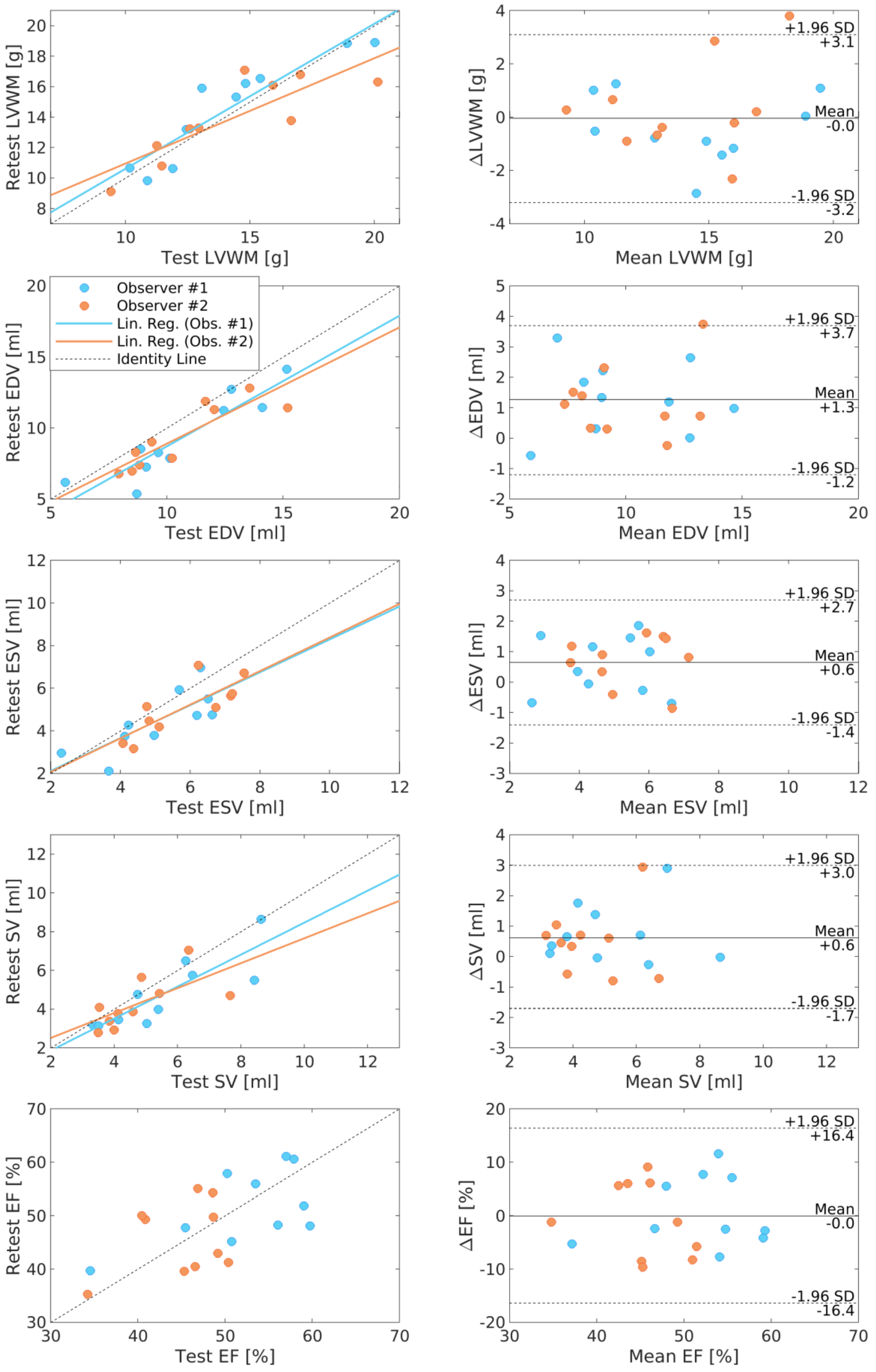

Supplementary Figure 4: Comparison of cine MRI and RT-MRI. Correlation and Bland-Altman plots of LVWM, EDV, ESV, SV and EF comparing cine MRI and RT-MRI of the second study time point (retest). RT-MRI significantly underestimated volumetric parameter by 20% to 40% when taking cine MRI as gold standard. However, regression analysis comparing estimated parameter by RT-MRI and cine MRI revealed a strong correlation. LVWM: Left Ventricular Wall Mass; EDV: End-Diastolic Volume; ESV: End-Systolic Volume; SV: Stroke Volume; EF: Ejection Fraction.

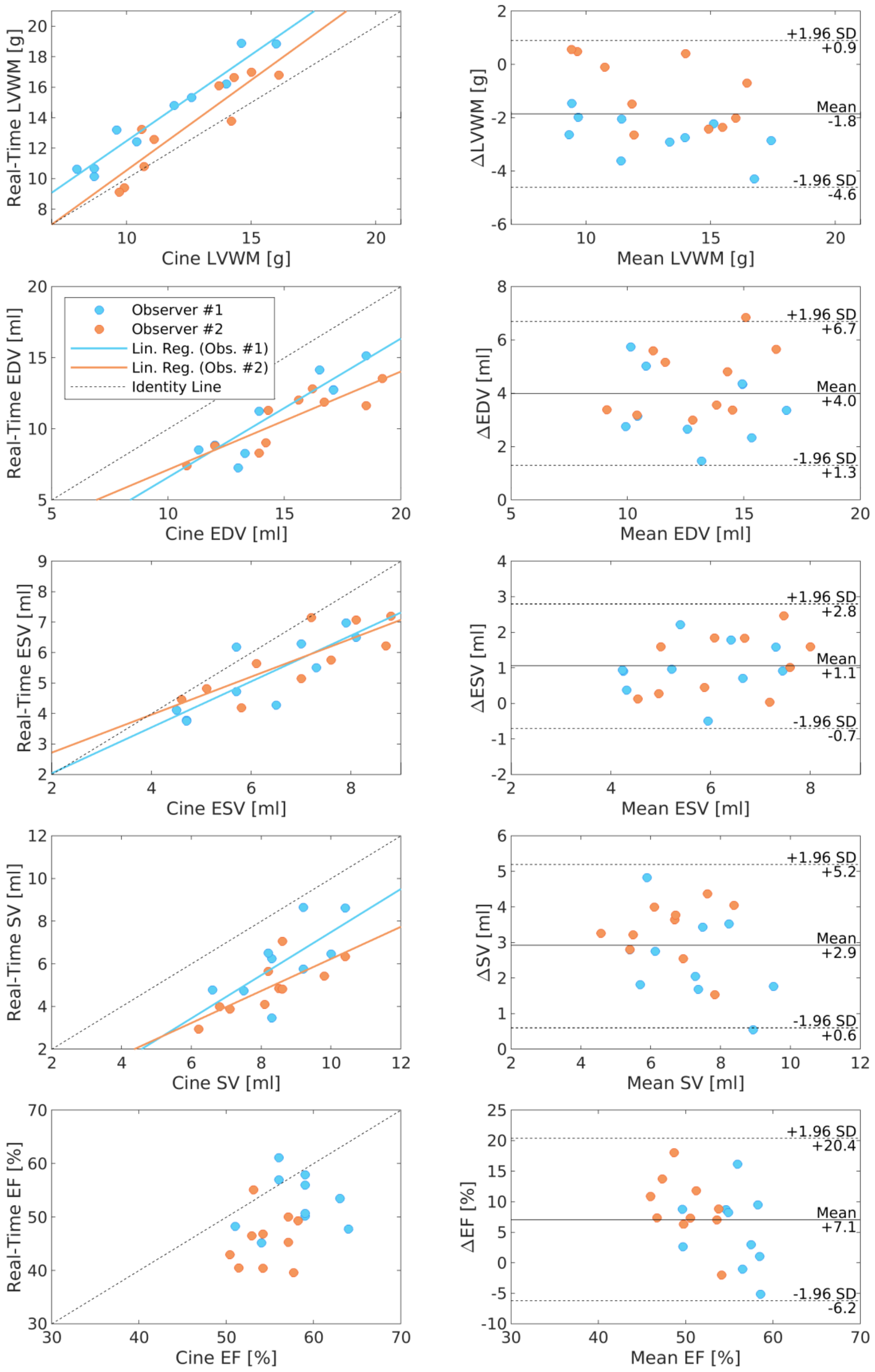

Supplementary Figure 5: Comparison of inspiratory and expiratory ventricular function. Correlation and Bland-Altman plots of LVWM, EDV, ESV and EF for inspiration and expiration obtained by RT-MRI of the second study time point (retest). Volumetric parameters (EDV, ESV and SV) were increased by at least 20% during inspiration compared to expiration. Additionally, a slight increase in EF was observed during inspiration. Nevertheless, a strong correlation between the two respiration phases exists. LVWM: Left Ventricular Wall Mass; EDV: End-Diastolic Volume; ESV: End-Systolic Volume; SV: Stroke Volume; EF: Ejection Fraction.

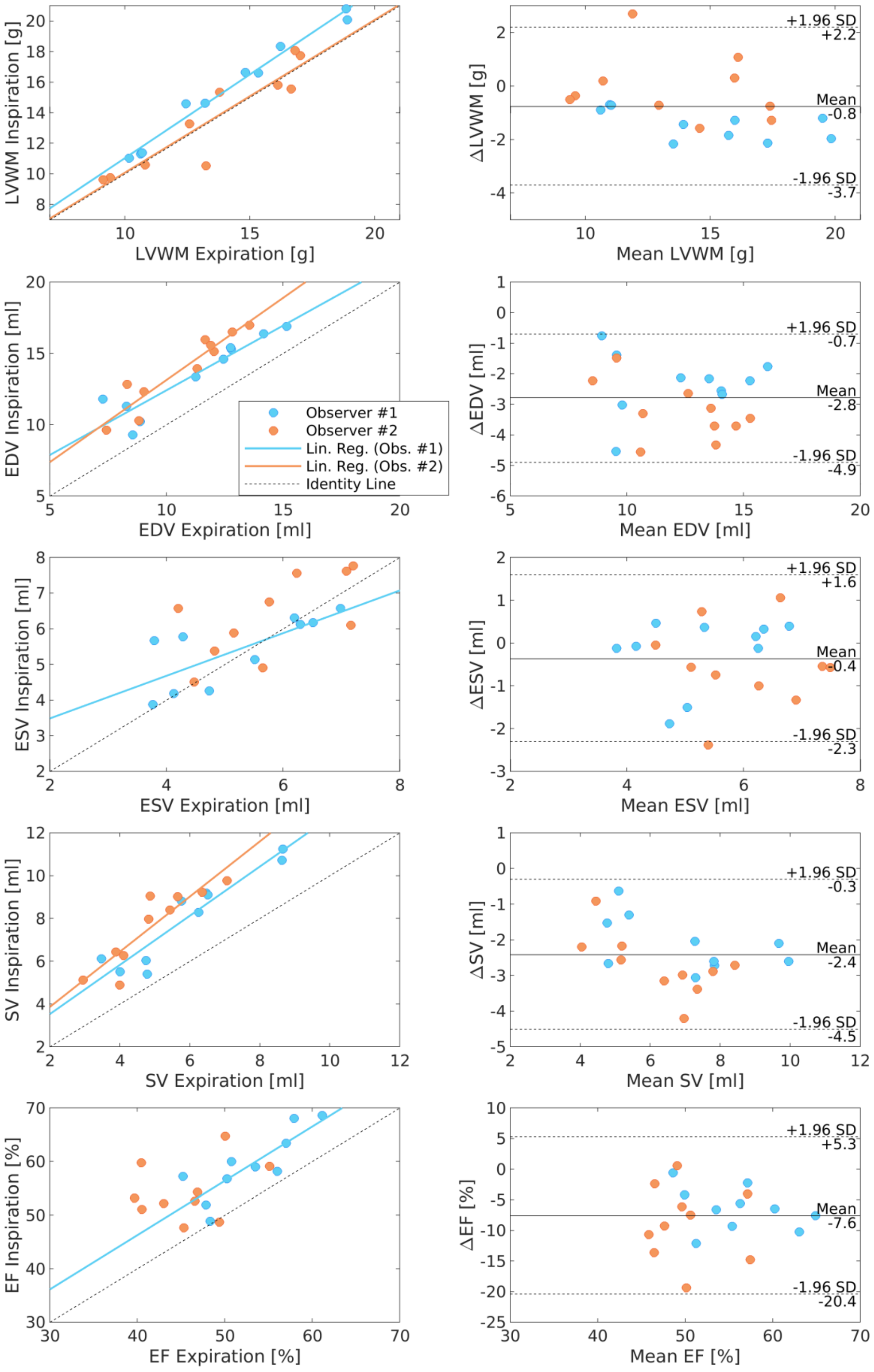

Supplement: Supplementary file 1 — Supplementary Information. [file 41598_2021_90106_MOESM1_ESM.pdf]
